# How job stress influences job performance among Chinese healthcare workers: a cross-sectional study

**DOI:** 10.1186/s12199-018-0758-4

**Published:** 2019-01-05

**Authors:** Jianwei Deng, Yilun Guo, Tengyang Ma, Tianan Yang, Xu Tian

**Affiliations:** 1 School of Management and Economics, Beijing Institute of Technology, Beijing, 100081 China; 2 Sustainable Development Research Institute for Economy and Society of Beijing, Beijing, 100081 China; 3 Institute of World Economics and Politics, Chinese Academy of Social Sciences, Beijing, 100732 China

**Keywords:** Challenge stress, Hindrance stress, Public service motivation, Job performance, Quality of healthcare, Big data

## Abstract

**Objectives:**

Public service motivation refers to the idea of commitment to the public service, pursuit of the public interest, and the desire to perform work that is worthwhile to society. This study investigates how challenge stress and hindrance stress influence job performance among healthcare workers in Chinese public hospitals. It has also examined the mediating effect of public service motivation.

**Methods:**

Data of 1594 healthcare workers were obtained from typical public hospitals in eastern, central, and western China. To test our hypotheses, we used descriptive statistical analysis, correlation analysis, structural equation modeling, and subgroup analysis to investigate the sample.

**Results:**

Challenge stress and hindrance stress were strongly correlated among healthcare workers in Chinese public hospitals (*β* = 0.59; *p* < 0.001). Challenge stress was significantly positively associated with public service motivation (*β* = 0.14; *p* < 0.001) and job performance (*β* = 0.13; *p* < 0.001). Hindrance stress was significantly negatively associated with public service motivation (*β* = − 0.27; *p* < 0.001) and job performance (*β* = − 0.08; *p* < 0.05). Public service motivation was directly positively associated with job performance (*β* = 0.58; *p* < 0.001), and it indirectly mediated the association between job stress and job performance.

**Conclusions:**

This study provides important empirical evidence on the effects of job stress and public service motivation on job performance among healthcare workers in Chinese public hospitals. Job performance may be raised by limiting hindrance stress, which provides moderate challenge stress and increases public service motivation.

## Introduction

Job stress can be defined as an individual’s response to external stimuli in the environment. Recent studies have indicated that job stress has a major effect on individual physiology, psychology, and behavior [1–3], e.g., job performance [4]. However, previous studies have mostly focused on the negative effects of job stress on performance [5], which argue that higher pressures can make individuals perform less effectively on tasks that call for tolerance and concentration [6], subsequently resulting in lower productivity and job quality [5]. Following the development of positive psychology, Cavanaugh indicates that job stress can be divided into two dimensions: hindrance stress and challenge stress [7]. Challenge stress refers to the job stress that individuals feel that they can overcome and that benefits their career development, such as job load, job responsibility, and time urgency. Meanwhile, hindrance stress refers to the stress that individuals feel they cannot overcome and that prevents their career development, such as role conflict, organizational politics, and work insecurity [8]. Although recent studies have paid attention to the positive effects of job stress, most of them have been theoretical and qualitative [9]. Therefore, this study examines the impact of hindrance stress and challenge stress on job performance among healthcare workers in Chinese public hospitals, which responds to the call for more studies on different types of job stress [10] and provides empirical evidence of the differences in the effects of these stresses on job performance.

Previous studies have shown that job stress can indirectly affect job performance through mediator variables. Job satisfaction is mainly considered as the mediator [11]. Indeed, few studies have examined the mediating effect of other specific motivation constructs that inspire individual performance [12]. For example, public service motivation (PSM) denotes the idea of commitment to the public service, pursuit of the public interest, and the desire to perform work that is worthwhile to society [13]. Unlike job satisfaction, which is often regarded as a work-related emotion variable, PSM is an intrinsic predisposition to do good for others and society [14]. Goleman indicates that emotions move us to goal achievement, while motives shape our desires and actions [15]. Previous studies have investigated the positive relationship between PSM, job satisfaction, and job performance [16, 17]. It has also been found that when individuals are exposed to higher pressures, they will become less sensitive to others which is manifested in a decrease of helping and an increase in aggression [6, 18]. Therefore, this study introduces PSM and explores the mediating role that it plays in the relationship between job stress and job performance, which contributes to the development of the study of the mediator between job stress and performance, and answers the call for more studies of the effect of PSM on other psychological constructs and work-related outcomes [14, 16].

**Fig. 1:**
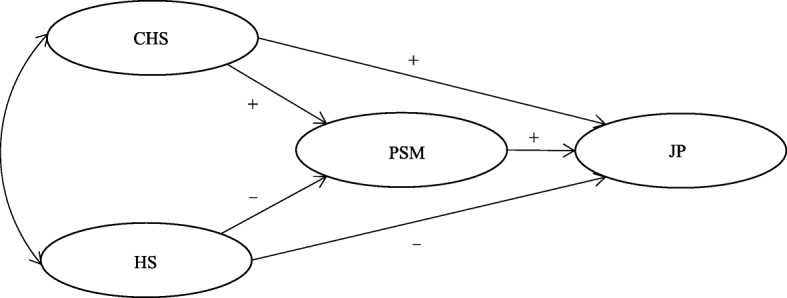
Proposed model of how challenge stress (CHS), hindrance stress (HS), and public service motivation (PSM) influence job performance (JP)

Overall, this study focuses on the relationship between job stress, PSM, and job performance (Fig. 1). Furthermore, this study provides empirical evidence for methods to alleviate pressure, improving performance and public service quality among Chinese healthcare workers.

## Materials and methods

### Data source

This study performed a cross-sectional analysis of data from 1594 healthcare workers (response rate 97.9%) in public hospitals from eastern, central, and western China in 2017, after obtaining ethical approval and informed consents. Table 1 shows the demographic characteristics of participants. Chinese public hospitals deliver more than 85% of hospital care and play a leading role in providing medical services [19]. Consequently, we chose public hospitals as our research focus. The ratio of public hospitals in eastern, central, and western China is 3.7:3.3:3 [19]. Therefore, we intended to use this ratio to randomly select 35, 24, and 24 public hospitals respectively. Limited by realistic conditions, we have actually selected 33, 14, and 19 public hospitals from these regions. Additionally, to guarantee data integrity and objectivity, this study randomly selected between 7 and 12% of healthcare workers in target hospitals through work identification numbers. Besides, to test convergent and divergent validity in our survey, we used similar and opposite questions in the questionnaire to examine whether our respondents provide with the expected answers. The participants will be deleted if their answers were not consistent.

**Table 1:** Demographic characteristics of the sample

Characteristics	Sample (*n* = 1594)	Percentage (%)
Sex		
Male	553	34.7
Female	1007	63.2
Age (years)		
− 25	129	8.1
25–30	442	27.7
31–35	390	24.5
36–40	223	14.0
41–45	142	8.9
46–50	139	8.7
51–55	87	5.5
56–60	33	2.1
Position		
Clinician	722	45.3
Nurse	523	32.8
Management	88	5.5
Medical technicians	143	9.0
Pharmacist	54	3.4
Education		
Under degree	75	4.7
Junior college	314	19.7
Undergraduate	763	47.9
Master’s	305	19.1
Doctorate	123	7.7
Title		
Trainee	665	41.7
Primary	581	36.4
Middle	205	12.9
Senior	80	5.0
Seniority (years)		
−3	313	19.6
3–5	335	21.0
6–10	347	21.8
11–20	303	19.0
20-	276	17.3
Department		
Physician	356	22.3
Surgery	296	18.6
Obstetrics/gynecology	190	11.9
Pediatrics	159	10.0
Chinese medicine	44	2.8
Emergency department/ICU	91	5.7
Oncology	31	1.9
Other clinical departments	100	6.3
Medical technology	147	9.2
Administration and logistics	84	5.3
Others	67	4.2

Demographic information was missing for a few participants (0.9–5.4%)

### Variables and instruments

Job stress was measured with the challenge and hindrance-related self-reported stress (C-HSS) scale of 11 items [7]. For example, the item, “The amount of time I spend at work” (Table 2) evaluates challenge stress. Meanwhile, the item, “The degree to which politics rather than performance affects organizational decisions” evaluates hindrance stress. The C-HSS scale uses a five-point Likert scale (1 = no stress; 5 = great stress). Higher values indicate greater job stress. In this study, the C-HSS scale was shown to have high reliability (*α* = 0.92–0.81).

**Table 2:** Mean (SD) for challenge stress (CHS), hindrance stress (HS), public service motivation (PSM), and job performance (JP) items

Variable	Item	Mean ± SD
Challenge stress (1–6)	CHS1. The number of projects and or assignments I have.	3.49 ± 0.82
	CHS2. The amount of time I spend at work.	3.52 ± 0.81
	CHS3. The volume of work that must be accomplished in the allotted time.	3.44 ± 0.88
	CHS4. Time pressures I experience.	3.48 ± 0.88
	CHS5. The amount of responsibility I have.	3.63 ± 0.89
	CHS6. The scope of responsibility my position entails.	3.50 ± 0.85
Hindrance stress (1–5)	HS1. The degree to which politics rather than performance affects organizational decisions.	2.87 ± 1.09
	HS2. The inability to clearly understand what is expected of me on the job.	2.35 ± 1.04
	HS3. The amount of red tape I need to go through to get my job done.	3.10 ± 1.03
	HS4. The lack of job security I have.	3.06 ± 1.13
	HS5. The degree to which my career seems “stalled.”	3.01 ± 1.03
PSM (1–5)	PSM1. Meaningful public service is very important to me.	3.98 ± 0.77
	PSM2. I am often reminded by daily events about how dependent we are on one another.	3.88 ± 0.80
	PSM3. Making a difference in society means more to me than personal achievements.	3.74 ± 0.85
	PSM4. I am prepared to make sacrifices for the good of society.	3.41 ± 0.96
	PSM5. I am not afraid to go to bat for the rights of others, even if it means I will be ridiculed.	3.45 ± 0.91
Job performance (1–4)	JP1. Quality of your performance.	3.80 ± 0.67
	JP2. Your productivity on the job.	3.86 ± 0.70
	JP3. How do you evaluate the performance of your peers at their jobs compared with yourself doing the same kind of work?	3.88 ± 0.68
	JP4. How do you evaluate the performance of yourself at your job compared with your peers doing the same kind of work?	3.86 ± 0.69

PSM was measured with a five-item scale developed by Coursey and Pandey [18]. For example, the item, “Making a difference in society means more to me than personal achievements” (Table 2) asks the respondents to rate their PSM on a scale from 1 (strongly disagree) to 5 (strongly agree). Higher values indicate greater PSM. In our research, this scale was shown to have high reliability (*α* = 0.84).

Job performance was measured with a four-item scale that was developed by Darwish [20]. For example, the questions “Quality of your performance” and “How do you evaluate the performance of yourself at your job compared with your peers doing the same kind of work?” (Table 2) ask the respondents to rate their job performance on a scale from 1 (no good) to 5 (very good). Higher values indicate greater job performance. The self-appraisal of job performance as an evaluation device had been adopted by others and yielded acceptable outcomes [21, 22]. This scale was shown to have high reliability (*α* = 0.86).

We also included individual characteristics, such as age, sex, education, job title, job experience, department, and seniority.

### Statistical analysis

This study used SPSS 20.0 and AMOS 20.0 for the statistical analyses, which included descriptive analysis, correlation analysis, and path analysis. In structural equation modeling (SEM), the latent variables included challenge stress, hindrance stress, PSM, and job performance. All of these indicators were evaluated to determine if the model fit the data well. For example, when a criterion such as a root mean square error of approximation is less than 0.08, and the normed fit index and comparative fit index are more than 0.90, then the model has good global fit [23]. Before imputing these indicators into the model, we used correlation analysis to determine the significance of the correlations between challenge stress, hindrance stress, PSM, and job performance. SEM can identify effect relationships among variables, which are classified as direct or indirect [24]. Finally, we used the Sobel test to examine the significance of mediated effects [25].

To determine if the standardized regression coefficients (*β*) differed by subgroup, we conducted subgroup analyses. To ensure that the subgroups were of equal size, the regions were classified as the eastern, central, and western. Hospital level was categorized as primary, secondary, and tertiary. Age was categorized as old (41 years or older), middle (31–40 years), and young (30 years or younger). Job title was classified as early career (trainee or entry-level worker) and mid/late career (mid-level or senior worker). Gender was categorized as male and female. Post was classified as physician, nurse, and other (e.g., management, medical technician, and pharmacist). Seniority was classified as less than 5 years (employed for less than 5 years) and greater than 5 years (employed for longer than 5 years).

## Results

### Mean (SD) of challenge stress, hindrance stress, PSM, and job performance

Table 2 shows the results, including mean (M) and SD for challenge stress, hindrance stress, PSM, and job performance items. The means for the challenge stress items were higher than those for hindrance stress items. The means of challenge stress ranged from 3.44 (SD = 0.88) to 3.63(SD = 0.89). The means of hindrance stress ranged from 2.35 (SD = 1.04) to 3.10 (SD = 1.03). The means for the five PSM items were high, and the range was moderate. The means ranged from 3.41 (SD = 0.96) to 3.98 (SD = 0.77). The means for the four job performance items were relatively high, and the range was minor. The means ranged from 3.80 (SD = 0.67) to 3.88 (SD = 0.68).

Tables 3 and 4 show that among different regions, hospital levels, and demographic characteristics of healthcare workers, the values of challenge stress, hindrance stress, PSM, and job performance are discrepant. For example, the challenge stress was significantly higher in eastern public hospitals (M = 3.54, SD = 0.70) and tertiary hospitals (M = 3.59, SD = 0.67). In terms of hindrance stress, healthcare workers who were middle-aged (M = 2.96, SD = 0.81), male (M = 2.97, SD = 0.88), and physicians (M = 2.94, SD = 0.83) had a higher level of hindrance stress. With respect to PSM, the participants who were old (M = 3.82, SD = 0.70) or working in tertiary hospitals (M = 3.74, SD = 0.69) performed at a higher level of PSM. The job performance was significantly higher among the participants who were old (M = 4.00, SD = 0.56), who had worked for over 5 years (M = 3.92, SD = 0.56), and who had a more senior job title (M = 4.00, SD = 0.56).

**Table 3:** The differences in challenge stress (CHS), hindrance stress (HS), public service motivation (PSM), and job performance (JP) between different regions and hospital levels

Variable	Region			*p*	Hospital level			*p*
	Eastern (*n* = 1153)	Central (*n* = 201)	Western (*n* = 240)		Primary (*n* = 284)	Secondary (*n* = 383)	Tertiary (*n* = 927)	
CHS	3.54 (0.70)	3.37 (0.65)	3.51 (0.83)	**	3.32 (0.72)	3.46 (0.79)	3.59 (0.67)	***
HS	2.89 (0.78)	2.84 (0.82)	2.83 (0.91)	–	2.86 (0.78)	2.81 (0.89)	2.91 (0.77)	–
PSM	3.71 (0.68)	3.59 (0.63)	3.72 (0.67)	–	3.65 (0.63)	3.60 (0.65)	3.74 (0.69)	***
JP	3.85 (0.58)	3.86 (0.53)	3.84 (0.59)	–	3.82 (0.56)	3.84 (0.56)	3.86 (0.59)	–

Numbers outside parentheses are mean values; numbers inside parentheses are SD values

***p < 0.001, **p < 0.01, *p < 0.05

**Table 4:** The differences in challenge stress (CHS), hindrance stress (HS), public service motivation (PSM), and job performance (JP) among the healthcare workers’ demographic characteristics

Variable	Age			*p*	Job title		*p*	Sex		*p*	Seniority		*p*	Post			*p*
	Young (*n* = 571)	Middle (*n* = 613)	Old (*n* = 401)		Early career (*n* = 1246)	Mid/late career (*n* = 285)		Men (*n* = 553)	Women (*n* = 1007)		Below 5 years (*n* = 648)	Above 5 years (*n* = 926)		Physicians (*n* = 722)	Nurses (*n* = 523)	Others (*n* = 285)	
CHS	3.39 (0.66)	3.60 (0.71)	3.54 (0.78)	***	3.49 (0.71)	3.62 (0.77)	*	3.54 (0.76)	3.50 (0.70)	–	3.39 (0.69)	3.60 (0.73)	*	3.60 (0.73)	3.45 (0.69)	3.42 (0.70)	***
HS	2.74 (0.78)	2.96 (0.81)	2.95 (0.81)	***	2.85 (0.80)	3.00 (0.83)	–	2.97 (0.88)	2.82 (0.76)	***	2.79 (0.81)	2.94 (0.80)	–	2.94 (0.83)	2.83 (0.77)	2.78 (0.81)	**
PSM	3.65 (0.66)	3.34 (0.66)	3.82 (0.70)	***	3.68 (0.67)	3.78 (0.72)	–	3.73 (0.70)	3.67 (0.66)	–	3.67 (0.65)	3.72 (0.69)	–	3.71 (0.68)	3.67 (0.66)	3.70 (0.68)	–
JP	3.76 (0.57)	3.83 (0.57)	4.00 (0.56)	***	3.82 (0.58)	4.00 (0.56)	*	3.87 (0.58)	3.83 (0.57)	–	3.75 (0.59)	3.92 (0.56)	**	3.85 (0.57)	3.83 (0.57)	3.90 (0.58)	–

Numbers outside parentheses are mean values; numbers inside parentheses are SD values

***p < 0.001, **p < 0.01, *p < 0.05

### Correlations between challenge stress, hindrance stress, PSM, and job performance

Table 5 shows the correlation coefficients (*r*) that explained the positive correlations between items within the same construct. Hindrance stress was significantly inversely correlated with job performance and PSM (*r* = − 0.13 to − 0.17). Challenge stress was significantly positively correlated with job performance (*r* = 0.06) but was not significantly correlated with PSM. There were also significant positive correlations both between PSM and job performance (*r* = 0.51), and between challenge stress and hindrance stress (*r* = 0.47).

**Table 5:** Intercorrelations between challenge stress (CHS), hindrance stress (HS), public service motivation (PSM), and job performance (JP) items

Variables	Mean (SD)	WP	CHS	HS	PSM
JP	3.85 (0.58)	1			
CHS	3.51 (0.72)	.06*	1		
HS	2.88 (0.81)	−.13**	.47**	1	
PSM	3.69 (0.67)	.51**	−.02	−.17**	1

*N* = 1594;**p < 0.01, *p < 0.05

## SEM

**Fig. 2:**
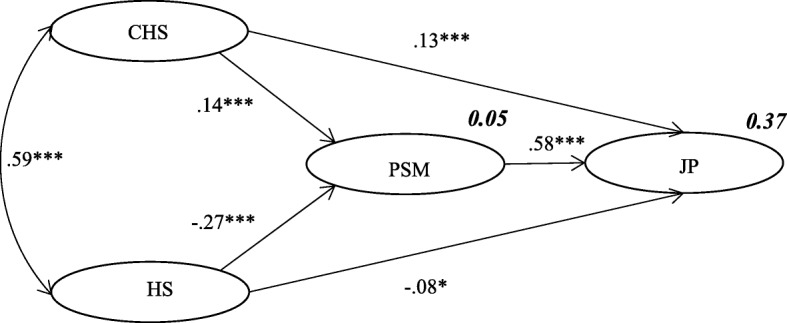
Final model illustrating how challenge stress (CHS), hindrance stress (HS), and public service motivation (PSM) influence job performance (JP) (numbers not in bold are standardized regression coefficients, and numbers in bold explain variability; chi-square, 974.925; degrees of freedom, 160, *p* < 0.001; root mean square error of approximation, 0.057; normed fit index, 0.943; comparative fit index, 0.952; ***p < 0.001, **p < 0.01,*p < 0.05)

In the final model, job stress was directly and significantly associated with PSM and job performance. Challenge stress was significantly positively associated with PSM (*β* = 0.14; *p* < 0.001) and job performance (*β* = 0.13; *p* < 0.001). Hindrance stress was significantly inversely associated with PSM (*β* = − 0.27; *p* < 0.001) and job performance (*β* = − 0.08; *p* < 0.05). PSM was significantly positively associated with job performance (*β* = 0.58; *p* < 0.001). There was also a direct positive association between challenge stress and hindrance stress (*β* = 0.59; *p* < 0.001). Challenge stress, hindrance stress, and PSM explained 37% of the variability in job performance. The criteria for fitness indicated that the revised model was more appropriate (Fig. 2).

We then noted significant indirect effects of challenge stress (Sobel *z* = 2.06; *p* < 0.05) and hindrance stress (Sobel *z* = − 6.07; *p* < 0.001) on job performance, which were both significantly mediated by PSM.

Tables 6 and 7 show the results of subgroup analyses. The impact of challenge stress on PSM was significant among the participants who were female (*β* = 0.20; *p* < 0.001), nurses (*β* = 0.27; *p* < 0.001), and working in eastern (*β* = 0.17; *p* < 0.001) or tertiary hospitals (*β* = 0.17; *p* < 0.001). Meanwhile, the hindrance stress did not significantly affect PSM among old (*β* = − 0.12; *p* > 0.05) and mid/late career healthcare workers (*β* = − 0.36; *p* > 0.05). Moreover, the effect of challenge stress on job performance was not significant among those working in the western region (*β* = 0.11; *p* > 0.05) and primary hospital healthcare workers (*β* = − 0.02; *p* > 0.05). The effect of hindrance stress on job performance was weaker than challenge stress, but between females (*β* = − 0.10; *p* < 0.05) and nurses (*β* = − 0.15; *p* < 0.05) hindrance stress significantly inversely affected job performance.

**Table 6:** Standardized regression coefficients (*β*) with *p* values for the components of subgroup analyses between different regions and hospital levels

Path	Region			Hospital level		
	Eastern (*n* = 1153)	Central (*n* = 201)	Western (*n* = 240)	Primary (*n* = 284)	Secondary (*n* = 383)	Tertiary (*n* = 927)
CHS to PSM	0.17***	− 0.07	0.13	− 0.01	0.07(0.392)	0.17***
HS to PSM	− 0.31***	0.08	− 0.37***	− 0.29***	− 0.28**	− 0.26***
PSM to JP	0.60***	0.68***	0.46***	0.70***	0.42***	0.59***
CHS to JP	0.13***	0.24*	0.11	− 0.02	0.27***	0.14***
HS to JP	− 0.10*	− 0.22*	0.03	0.05	− 0.27***	− 0.05
CHS to HS	0.59***	0.67***	0.45***	0.47***	0.56***	0.60***

**Table 7:** Standardized regression coefficients (*β*) with *p* values for the components of subgroup analyses among the healthcare workers’ demographic characteristics

Path	Age			Job title		Sex		Seniority		Post		
	Young (under 30 years, *n* = 571)	Middle (31–40 years, *n* = 613)	Old (over 41 years, *n* = 401)	Early career (*n* = 1246)	Mid/late career (*n* = 285)	Men (*n* = 553)	Women (*n* = 1007)	Below 5 years (*n* = 648)	Above 5 years (*n* = 926)	Physicians (*n* = 722)	Nurses (*n* = 523)	Others (*n* = 285)
CHS to PSM	0.12	0.20***	0.05	0.17***	0.002	0.08	0.20***	0.07	0.16***	0.08	0.27***	0.02
HS to PSM	− 0.36***	− 0.32***	− 0.12	− 0.33***	− 0.08	− 0.23***	− 0.33***	− 0.33***	− 0.24***	− 0.22***	− 0.41***	− 0.13
PSM to JP	0.52***	0.60***	0.62***	0.56***	0.65***	0.58***	0.58***	0.57***	0.60***	0.59***	0.56***	0.57**
CHS to JP	0.07	0.15**	0.15*	0.12**	0.15*	0.15**	0.12**	0.07	0.15***	0.13**	0.090	0.17*
HS to JP	− 0.12	− 0.05	− 0.10	− 0.07	− 0.11	− 0.07	− 0.10*	− 0.06	− 0.11*	− 0.01	− 0.15*	− 0.10
CHS to HS	0.59***	0.50***	0.62***	0.57***	0.56***	0.58***	0.57***	0.52***	0.59***	059***	0.58***	0.47**

## Discussion

### Main finding of this study

This study conducted a survey of 1594 Chinese healthcare workers from public hospitals to investigate the relationship between job stress, PSM, and job performance. Overall, the main findings are as follows.

Our first main finding is that the effect of different types of job stress on PSM is significant but diverse. As expected, hindrance stress adversely affects PSM. Previous studies have shown that hindrance stress could increase burnout [26], but very few studies have investigated the relationship between hindrance stress and PSM. Therefore, this study further confirms the negative impact of job stress, especially hindrance stress, on individuals and organizations. Meanwhile, this study enriches the antecedents research of PSM from the perspective of external environmental stimulus. Interestingly, our subgroup analysis showed that hindrance stress has a very strong inhibitory effect on PSM among Chinese healthcare workers in public hospitals, which may relate to the complexity of the healthcare environment. First, job stress among Chinese healthcare workers is extremely high [27] and they also have health problems at work [28], which further affect their emotional well-being and behaviors [29]. Second, Chinese healthcare workers are extremely overworked and they earn lower incomes compared to their counterparts in Europe and the USA [8]. The imbalance between giving and gain could restrain the PSM of Chinese healthcare workers [30]. Finally, the frequent occurrence of violence against Chinese healthcare workers leads to a lack of job security [31, 32]. Unfair promotion and career advancement in Chinese public hospitals also hinders career development among Chinese healthcare workers [33]. All of these factors aggravate hindrance stress and limit PSM.

In this study, we found that challenge stress is significantly positively associated with PSM among Chinese healthcare workers, which is in agreement with most previous studies. Therefore, this study further explores the effect of challenge stress in Chinese public hospitals and it provides empirical support for studies of the positive impact of job stress [34]. Interestingly, our subgroup analysis showed that the impact of challenge stress on PSM was significant among those participants who were working in eastern or tertiary hospitals, females, and nurses. The challenge stress of healthcare workers was significantly higher in eastern public hospitals and tertiary hospitals. Eastern region has more adequate medical resources as the most economically developed region in China [19]. However, based on this, the competition between hospitals in the eastern region is also increasing [35]. In addition, the reform of public hospitals has been a top priority since 2009 and, therefore, tertiary hospitals that play a dominant role in health delivery need to establish an effective and efficient system to deliver more cost-effective and higher-quality services [36]. In this case, it is necessary for hospitals in the eastern region and tertiary hospitals to continuously stimulate healthcare workers to improve the quality of public services and meet their development needs, such as job titles, honors, and benefits [37]. Thus, to achieve better career development, the increase in competition among healthcare workers has stimulated the positive effect of challenge stress. Nevertheless, the impact of challenge stress on PSM was significant among females and nurses, which may relate to staff composition and the nurses’ job characteristics. Nurses are mostly women, and the whole nursing process is actually individualized care [38]. Driven by high work responsibilities through longer and more frequent contact with patients, nurses will tend to show a higher level of compassion and caring for patients, and this will promote their PSM.

Our second key finding is that PSM significantly and positively affects job performance among Chinese healthcare workers. Although many studies have found that PSM has a positive impact on individuals and organizations [39, 40], recent studies suggest that PSM also has a dark side [41, 42]. Therefore, the impact of PSM on individuals remains controversial. We suggest that the different work environments lead to different reactions. Furthermore, some studies have found that the positive effects of PSM can change over time, particularly in hostile working conditions [39]. Consequently, this study examined the effect of PSM in Chinese public hospitals and supports the view that PSM has a positive impact on individuals and organizations. Healthcare workers with high levels of PSM can fit better with the values of the hospitals. They will then perform better to achieve their own values. This finding shows that to develop the positive effect of PSM, managers should try to ameliorate the working environment and psychological status of healthcare workers, such as reducing workplace violence [32] and demonstrating more social support [43].

Our third key finding is that challenge stress positively affects job performance while hindrance stress inversely affects job performance. This is in agreement with most previous studies, which have shown that challenge stress has a positive effect on job performance due to the individuals’ positive response and hindrance stress as an excessive demand leads to poor job performance [8]. However, this study has shown that the effect of hindrance stress on job performance is weaker than the effect of challenge stress, which may be caused by the special nature of the work done by healthcare workers. As providers of medical services, the work efficiency and quality of healthcare workers are directly linked to public service quality, and even related to public health and safety. Therefore, although hindrance stress has a negative impact on job performance among healthcare workers, the effect is weaker than challenge stress. This finding suggests that policymakers should implement differential intervention pressure measures, focusing on the negative impact of hindrance stress, for example, by simplifying work processes and reforming pay structures. In addition, policymakers should fully utilize the positive effect of challenge stress through the establishment of reasonable work intensity [14] and reasonable shift work. However, it should be noted that excessive challenge stress, such as overload, may have negative effects on organizations and individuals [44]. Therefore, future studies should investigate ways to control challenge stress within a reasonable range. They should also define the critical points between challenge stress and hindrance stress. In addition, a previous study has argued that because a stressful workplace will not be easy to change, it is essential for healthcare workers to learn how to cope with job stress [29]. Given that job stress among Chinese healthcare workers is very high, policymakers should provide relevant training and guidance for healthcare workers through employee assistance programs.

Finally, this study finds that PSM indirectly mediates the association between job stress and job performance among Chinese healthcare workers, which can be explained by social exchange theory. In this study, the healthcare workers expect that lower hindrance stress and moderate challenge stress will promote career development, which should be provided by the hospitals. The public hospitals need enthusiastic service and good performance, which can be provided by the healthcare workers. Therefore, on the basis of the “reciprocity principle” [45], healthcare workers exchange low hindrance stress and moderate challenge stress by showing high levels of PSM and job performance. This study further explores the impact of job stress on job performance from the perspective of the individual internal driving force. It also enriches the mediating studies between job stress and job performance. According to our findings, policymakers should fully consider the effect of PSM. In particular, managers should optimize their staff recruitment and selection systems [46] to absorb individuals with a high level of PSM into the hospitals. They could also strengthen the organizational culture to achieve the best fit between individual and organizational values.

### Limitations and future directions

This study has several limitations that deserve attention. First, this study was conducted through a cross-sectional survey. The relationship between challenge stress, hindrance stress, PSM, and job performance cannot be assumed to be causal and, therefore, it should be tested in future longitudinal studies. Second, we only recruited Chinese healthcare workers from public hospitals, and we excluded those healthcare workers from private hospitals. This restricts the generalizability and robustness of our conclusions. Although PSM is viewed as particularly salient in public organizations [47], it can also be found in the private sector [48]. Consequently, in the future, healthcare workers in private hospitals should be investigated to develop our hypothesis and models. Third, job performance was self-reported in our study. Consequently, the data might be subjective and positive. Future studies should add objective data into the study design to explore whether job stress or PSM affect job performance. Fourth, this study only explored the impact of job stress and PSM on job performance. This meant that we ignored other work-related outcomes, such as public service quality and organizational citizenship behavior. Therefore, it is recommended that future studies empirically investigate the relationship between job stress, PSM, and other work behaviors.

## Conclusion

Although job stress has been a subject of constant concern, there is a lack of empirical research on the effects of different types of job stress on productivity-related outcomes [26]. Through a cross-sectional analysis of 1594 Chinse healthcare workers from public hospitals, this study has found that healthcare workers are the key to improving public service quality. However, they generally suffer from high levels of job stress and this is likely to inhibit their PSM and then leads to poor job performance. To improve job performance and public service quality, public hospital administrators should pay attention to the relationship between challenge stress, hindrance stress, PSM, and job performance. They should also focus on targeting interventions to improve work conditions and maintain a high level of PSM. In addition, it is important to help healthcare workers to effectively cope with hindrance stress, and hospitals should promote personal professional development.

## Abbreviations

PSM: Public service motivation
SEM: Structural equation modeling
